# Text Message Analysis Using Machine Learning to Assess Predictors of Engagement With Mobile Health Chronic Disease Prevention Programs: Content Analysis

**DOI:** 10.2196/27779

**Published:** 2021-11-10

**Authors:** Harry Klimis, Joel Nothman, Di Lu, Chao Sun, N Wah Cheung, Julie Redfern, Aravinda Thiagalingam, Clara K Chow

**Affiliations:** 1 Faculty of Medicine and Health, Westmead Applied Research Centre The University of Sydney Westmead Australia; 2 Department of Cardiology Westmead Hospital Westmead Australia; 3 Sydney Informatics Hub The University of Sydney Camperdown Australia; 4 Department of Endocrinology Westmead Hospital Westmead Australia; 5 Western Sydney Integrated Care Program Sydney Australia

**Keywords:** mHealth, machine learning, chronic disease, cardiovascular, text messaging, SMS, digital health, mobile phone, engagement, prevention

## Abstract

**Background:**

SMS text messages as a form of mobile health are increasingly being used to support individuals with chronic diseases in novel ways that leverage the mobility and capabilities of mobile phones. However, there are knowledge gaps in mobile health, including how to maximize engagement.

**Objective:**

This study aims to categorize program SMS text messages and participant replies using machine learning (ML) and to examine whether message characteristics are associated with premature program stopping and engagement.

**Methods:**

We assessed communication logs from SMS text message–based chronic disease prevention studies that encouraged 1-way (SupportMe/ITM) and 2-way (TEXTMEDS [Text Messages to Improve Medication Adherence and Secondary Prevention]) communication. Outgoing messages were manually categorized into 5 message intents (informative, instructional, motivational, supportive, and notification) and replies into 7 groups (stop, thanks, questions, reporting healthy, reporting struggle, general comment, and other). Grid search with 10-fold cross-validation was implemented to identify the best-performing ML models and evaluated using nested cross-validation. Regression models with interaction terms were used to compare the association of message intent with premature program stopping and engagement (replied at least 3 times and did not prematurely stop) in SupportMe/ITM and TEXTMEDS.

**Results:**

We analyzed 1550 messages and 4071 participant replies. Approximately 5.49% (145/2642) of participants responded with *stop*, and 11.7% (309/2642) of participants were engaged. Our optimal ML model correctly classified program message intent with 76.6% (95% CI 63.5%-89.8%) and replies with 77.8% (95% CI 74.1%-81.4%) balanced accuracy (average area under the curve was 0.95 and 0.96, respectively). Overall, *supportive* (odds ratio [OR] 0.53, 95% CI 0.35-0.81) messages were associated with reduced chance of stopping, as were *informative* messages in SupportMe/ITM (OR 0.35, 95% CI 0.20-0.60) but not in TEXTMEDS (for interaction, *P*<.001). *Notification* messages were associated with a higher chance of stopping in SupportMe/ITM (OR 5.76, 95% CI 3.66-9.06) but not TEXTMEDS (for interaction, *P*=.01). Overall, *informative* (OR 1.76, 95% CI 1.46-2.12) and *instructional* (OR 1.47, 95% CI 1.21-1.80) messages were associated with higher engagement but not *motivational* messages (OR 1.18, 95% CI 0.82-1.70; *P*=.37). For *supportive* messages, the association with engagement was opposite with SupportMe/ITM (OR 1.77, 95% CI 1.21-2.58) compared with TEXTMEDS (OR 0.77, 95% CI 0.60-0.98; for interaction, *P*<.001). *Notification* messages were associated with reduced engagement in SupportMe/ITM (OR 0.07, 95% CI 0.05-0.10) and TEXTMEDS (OR 0.28, 95% CI 0.20-0.39); however, the strength of the association was greater in SupportMe/ITM (for interaction *P*<.001).

**Conclusions:**

ML models enable monitoring and detailed characterization of program messages and participant replies. Outgoing message intent may influence premature program stopping and engagement, although the strength and direction of association appear to vary by program type. Future studies will need to examine whether modifying message characteristics can optimize engagement and whether this leads to behavior change.

## Introduction

Mobile health (mHealth) is increasingly being used to support individuals with chronic diseases in novel ways that leverage the mobility and capabilities of mobile phones [1]. Owing to the global ubiquity of mobile phones, the predominant perceived benefit of mHealth is the potential to rapidly scale and bridge geographical, financial, and cultural access barriers to health care and, thus, provide population health benefits. SMS text message–based interventions, in particular, may have greater potential for reaching lower-income groups and those with poor health compared with smartphone app–based interventions [2]. There is evidence suggesting that SMS text message–based interventions can result in improvement in multiple behavior-related risk factors, including smoking, physical activity, blood pressure, weight, and diabetes mellitus [3-7]. However, intervention effect sizes are modest, and the duration of the effect is uncertain [8].

Understanding how SMS text message–based program content affects participant replies may aid in optimizing future SMS text message–based programs. For example, SMS text message content that have been associated with premature program withdrawal can be avoided, and content that participants engage most with can be used more frequently. Engagement with mHealth programs has been considered an important factor in their effectiveness [9] and has been most commonly defined by frequency (ie, how often contact is made) and dropouts [10,11]. However, there are knowledge gaps, including how to maximize engagement with mHealth programs and whether mHealth content affects engagement. Incorporating instructional behavior change techniques within the mHealth program content, such as action plans [12] and setting goals [13], may help promote and sustain healthy lifestyle behaviors in patients with chronic diseases, including cardiovascular disease (CVD). The degree of interactivity (ie, 1-way vs 2-way flow of information) may also affect engagement [14], although, to date, it is poorly understood.

Our team has previously developed and supported patients with SMS text message–based mHealth programs who have chronic diseases, including CVD [15-18], diabetes [17], renal disease [19], and chronic obstructive pulmonary disease [18]. Across our programs, we developed a database of different SMS text messages and participant replies. The first step in understanding the complex interaction between mHealth content and participant replies is to categorize message content into *themes* (ie, groups of messages that share common features, such as reporting struggle, or common goals, such as motivation). To do this manually is time consuming, not practicable, and would limit scalability. As a solution, machine learning (ML) models can be trained to automatically categorize text [20], potentially saving time and resources, and is reproducible, improving the scalability of the program. In addition, this would allow contextualized assessment of participant replies (ie, assessment of replies in context with the program message participants are replying to) and engagement. Therefore, the aims of this research are (1) to develop ML models to categorize program SMS text messages and participant replies and (2) to examine whether message characteristics were associated with premature program stopping and engagement.

## Methods

### Study Population

We analyzed our combined communication logs from 3 Australian SMS text message–based digital health programs (SupportMe, ACTRN12616001689460 [17]; TEXTMEDS (Text Messages to Improve Medication Adherence and Secondary Prevention), ACTRN12613000793718 [16]; and ITM ACTRN12616001167459 [18]). In brief, with respect to the original studies (Table 1), SupportMe was a 6-month single-blinded, multicenter randomized controlled trial (RCT) for participants from community and hospital settings with a history of CVD or type 2 diabetes mellitus, and the primary outcome was systolic blood pressure at 6 months [17]. The TEXTMEDS study was a 12-month single-blinded, multicenter RCT delivered to patients following an acute coronary syndrome, with the primary end point being the percentage of patients who are adherent to cardioprotective medications [16]. ITM was a 6-month multicenter, single-blinded RCT targeting patients attending cardiac or pulmonary rehabilitation, with the primary outcome being exercise capacity, as measured by the 6-minute walk test [18]. For both SupportMe [17] and ITM [18], participants were not encouraged to reply to messages they received (ie, 1-way communication), although they were still able to reply, and replies were monitored. TEXTMEDS [16] encouraged replies from participants (ie, 2-way communication) and included the opportunity to liaise with a health counselor. Analysis of SupportMe and ITM were grouped together and referred throughout the manuscript as SupportMe/ITM, as both programs were similar (1-way communication not involving a health counselor) and recruited community and hospital participants with stable chronic conditions compared with TEXTMEDS (2-way with a health counselor and following a hospital admission for an acute cardiovascular event). Despite differences in the setting that participants were recruited from (ie, community vs hospital), the programs were delivered during the stable chronic phase of the illness (ie, as an outpatient) to deliver appropriate secondary prevention recommendations. The TEXTMEDS and SupportMe studies had primary ethics approval from the Western Sydney Local Health District Human Research Ethics Committee (TEXTMEDS: HREC2012/12/4.1 (3648) AU RED HREC/13/WMEAD/15; SupportMe: AU RED HREC/16/ WMEAD/331). The ITM study received primary ethics approval from the Sydney Local Health District Hospital Human Research Ethics Committee and associated governance committees at the sites.

**Table 1:** SMS text message–based prevention programs for metabolic disease.

Project	Duration	2-way communication encouraged^a^	Population	Recruitment number					Number of replies
				Total	Lost to follow-up	Withdrawn consent	Deaths		
TEXTMEDS^b^ [16]	12 months	Yes	CVD^c^ (recruited from hospital post-ACS^d^)	1424 (716 in intervention arm; 1:1 allocation)	39 (9 in intervention)	6 (1 in intervention)	15 (10 in intervention)	2356	
ITM (support for patients with respiratory disease and CVD via integrated SMS text messaging) [18]	6 months	No	CVD and respiratory disease (recruited from community with one or more chronic conditions)	316 (236 in intervention arm, 80 in control arm; 3:1 allocation)	26 (22 in intervention)	19 (12 in intervention)	4 (3 in intervention)	417	
SupportMe (SMS text messaging support for patients with chronic disease) [17]	6 months^e^	No	CVD and diabetes (recruited from community and hospital with one or more chronic conditions)	902 (454 in intervention arm; 1:1 allocation)	15 (9 in intervention)	7 (4 in intervention)	9 (5 in intervention)	1298	

^a^Two-way communication was possible with all the included SMS text message–based programs but only encouraged for TEXTMEDS (Text Messages to Improve Medication Adherence and Secondary Prevention).

^b^TEXTMEDS: Text Messages to Improve Medication Adherence and Secondary Prevention.

^c^CVD: cardiovascular disease.

^d^ACS: acute coronary syndrome.

^e^A total of 7 patients in SupportMe at the conclusion of the 6-month intervention continued into a 6-month maintenance phase, which consisted of receiving texts at half the original frequency.

### Developing ML Models to Characterize Text Messages

A total of 2 health professionals (HK and Anu Indrawansa, Westmead Hospital, Sydney, New South Wales, Australia) manually categorized all outgoing program SMS text messages in our SMS text message bank, all replies from SupportMe/ITM, and 829 TEXTMEDS replies.

Outgoing SMS text message intent (Textbox 1) was categorized as *informative* (provides health facts or education), *instructional* (provides tips or recommendations), *motivational* (provides feedback to encourage healthy behavior), *supportive* (provides contact details or referral to support groups or websites), and *notification* (notifies the patient about matters that are not educational, such as the welcome and exit messages). These message intent categories were chosen as they align with the dominant behavioral techniques used to develop the SMS text message bank, that is, provision of information about behavior health link and consequences (information-motivation- behavioral skills model), provision of instructions (social cognitive theory), provision of general encouragement (social cognitive theory), and relapse prevention (support to manage potential failure) [21].

**Example SMS text messages from each message intent category.**

**Informative**

- “[person_name] by switching from full fat to low fat milk in tea & coffee you could remove 1 kg of saturated fat from your diet a year!” [TEXTMEDS; Text Messages to Improve Medication Adherence and Secondary Prevention]
- “There are many ways to increase your activity levels [person_name]. Try Tai Chi, pilates, gardening, yoga or dancing” [ITM]
- “Did you know a blood test called HbA1c measures your average blood sugar over the last 3 months? Ask your doctor for a check every 3-6 months” [SupportMe]

**Instructional**

- “Cardiac drugs are safe but if you have any side effects discuss with your Dr - there are many medication options [person_name].” [TEXTMEDS]
- “If you are feeling more breathless than usual, try to relax, rest and practice your breathing techniques” [ITM]
- “[person_name], use up vegies by mixing them with herbs, spices & water to cook up a hearty soup” [SupportMe]

**Supportive**

- “Are you having a good week [person_name]? Just reminding you that you can text us if we can be of help” [TEXTMEDS]
- “Staying calm when you are breathless really helps. Is there someone in your household who can help you stay calm when you feel uptight?” [ITM]
- “Hi [person_name], you may need extra carbohydrates before, during, or after exercise to prevent low blood sugars - discuss with your healthcare team” [SupportMe]

**Motivational**

- “Dont worry [person_name] if you have a bad day. Remember that there is another chance tomorrow to choose the healthy option.” [TEXTMEDS]
- “Hi [person_name], when you are quitting smoking - if you have a bad day, don’t worry & keep trying” [ITM]
- “Hi [person_name], did you exercise today?” [SupportMe]

**Notification**

- “Hi [person_name], you are now halfway through TEXTMEDS. Soon we will ring to check how you are, but don’t tell us you have been receiving messages” [TEXTMEDS]
- “Hi [person_name], welcome to the ITM study. We hope you enjoy the messages. Respond STOP to opt out” [ITM]
- “Hi [person_name], welcome to the SupportMe study. You are in the group that will not receive regular messages. We will contact you at 6 months” [SupportMe]

Participant replies were categorized as follows: *stop* (replies indicating participants wish to stop receiving further messages), *thanks* (replies showing appreciation), *question* (replies prompting a response from the health counselor or research team), *reporting healthy* (replies indicating participants are complying with health recommendations), *reporting struggle* (replies indicating difficulties with complying with health recommendations), *general comment* (general replies regarding the program or health), and *other* (replies not fitting the above categories or not study related, for example, blank messages, inadvertent replies, and invalid numbers).

If an SMS text message could belong to 2 different categories or *intents* (eg, *informative* and *instructional*), the majority category or *intent* was favored.

Before developing the ML models, the outgoing messages were grouped into *clusters* of duplicate and highly similar messages in an attempt to support the automatic classification of related and novel future messages (Lancaster Stemmer method [22]). The similarity between 2 messages was measured by calculating the proportion of tokens (eg, words or punctuations) that were common between them (Jaccard similarity) [23]. We used 0.5 as the similarity threshold (ie, any 2 texts with a Jaccard similarity score >0.5 were put into the same cluster). Each ML model was tasked with using only the given *input features* to classify each item into a single best *output category.* For the program message (ie, outgoing messages) predictive model, the input features were the SMS text message, and the output category was the message intent. For the participant replies, the input features were the reply message (DistilBERT embeddings as detailed below) and the message intent of the outgoing message (1-hot encoded), and the output categories were the participant reply categories listed above.

Each ML model was created using a DistilBERT model pretrained on the Toronto Book Corpus and full English Wikipedia to encode word meaning and sentence structure (*distilbert-base-uncased*) [24]. We then applied an L2-regularized logistic regression model to weigh the 768 real-valued features produced by DistilBERT, generating a classification. We used grid search with 10-fold cross-validation to optimize the model hyperparameters (*inner cross-validation*). The selected hyperparameters were those that maximized balanced accuracy, which is defined as the mean sensitivity across all classes. For evaluation, we performed this procedure within a 10-fold cross-validation (*outer cross-validation*) using a technique known as nested cross-validation. Specifically, for each fold, 10% of all data were held out for testing, and within each of these, a 10-fold cross-validation was applied again with a similar 90% training to 10% validation split to select the best hyperparameters. To avoid biases in evaluation, we constrained the sampling of held out data sets such that near-identical outgoing messages (ie, those in the same Jaccard similarity cluster) could not appear in both training and test data set intent classifications. Similarly, for reply classification, we controlled for idiosyncratic language in the cross-validation procedure by ensuring that no participant replies appeared in both the training set and its corresponding test set.

### Associations of Message Characteristics to Premature Program Stopping and Engagement

We assessed associations using univariate logistic regression between outgoing message characteristics (outgoing message intents: informative, instructional, motivational, supportive, and notification) to the outcomes (1) reply type *stop* and (2) engagement. Engagement is difficult to define in the setting of SMS text message–based prevention programs; however, the frequency of participant replies does give a quantifiable measure of engagement as it reflects that the participant is repeatedly interacting with the program. Thus, engagement in this study was defined as a patient who replied at least 3 times and did not prematurely withdraw from the study or stop the intervention. An interaction analysis was performed to assess whether the associations were affected by program type (SupportMe/ITM vs TEXTMEDS) as the programs encouraged different levels of interaction (ie, 1-way vs 2-way communication; Table 1). To do this, univariate logistic regression models (between outgoing message characteristics to the outcomes as described above) were used, incorporating program type (SupportMe/ITM and TEXTMEDS) as the interaction term.

### Statistical Analysis

ML model accuracy was assessed using balanced accuracy, the area under the receiver operating characteristic (ROC) curve, and multiclass classification evaluators. This was done using the Scientific Python stack (Scikit-learn 0.22, Pandas 1.1, and Matplotlib 3.3) on Python 3.7 (Python Software Foundation). Associations of message characteristics to program termination and engagement were examined using SPSS Statistics (version 26.0; SPSS Inc). Chi-square tests of independence were performed to determine associations between the ML-derived program message intent and the outcomes (1) reply type *stop* or (2) engagement. Two-sided *P*<.05 were considered statistically significant unless otherwise stated.

## Results

### Descriptive Analyses of Program Messages and Replies

We analyzed a total of 1550 program messages and 4071 participant replies. The total number of patients in each group for each study and the received responses are shown in Table 1. Approximately, 30.01% (793/2642) of participants replied at least once, and 5.83% (154/2642) sent a *stop* reply type to opt out of future messages. For SupportMe/ITM, 32.35% (394/1218) participants replied to at least one message (384/394, 97.5% were from the intervention arm and 10/394, 2.5% were from the control arm). Of these, 20.8% (82/394) sent a *stop* reply type, of which 7 were from the control group. For TEXTMEDS, 28.02% (399/1424) of participants replied to at least one message (all were from the intervention arm), of which 18% (72/399) sent a *stop* reply type. For TEXTMEDS, most replies were in response to *supportive* messages (794/2356, 33.7% of all replies), and for SupportMe/ITM, it was in response to *informative* messages (661/1715, 38.54% of all replies; Multimedia Appendix 1). The majority reply type was *general comment* for both TEXTMEDS (635/2356, 26.95%) and SupportMe/ITM (574/1715, 33.47%; Multimedia Appendix 2). The types of responses elicited by each message intent for SupportMe/ITM and TEXTMEDS are detailed in the Multimedia Appendices 3 and 4.

**Figure 1:**
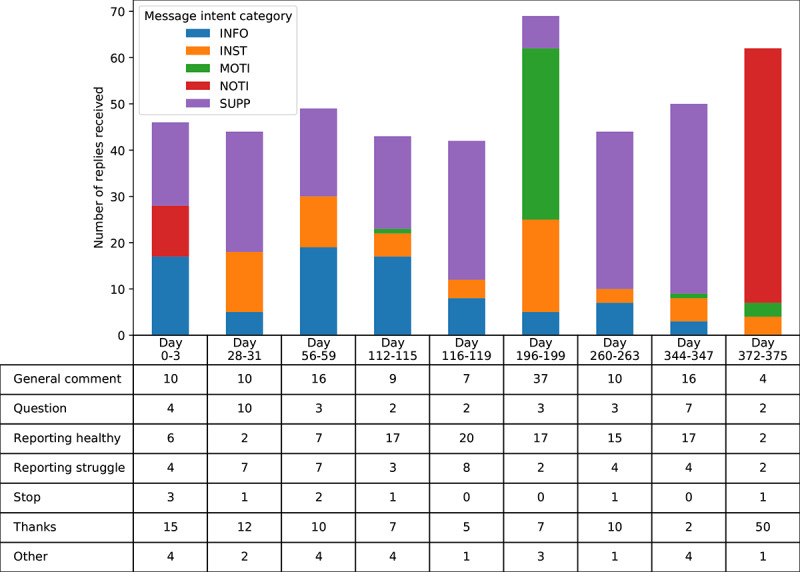
Distribution of participant reply categories by program message intent during the 12-month TEXTMEDS (Text Messages to Improve Medication Adherence and Secondary Prevention) program. The 9 peak periods (4-day duration each) were defined as those which received >40 replies within each peak period. INFO: Informative; INST: Instructional; MOTI: Motivational; NOTI: Notification; TEXTMEDS: Text Messages to Improve Medication Adherence and Secondary Prevention; SUPP: Supportive.

Approximately 11.43% (302/2642) of participants met our definition of being engaged. For SupportMe/ITM, 8.05% (98/1218) of participants were engaged and contributed to 78.6% (1348/1715) of the total replies in SupportMe/ITM. For TEXTMEDS, 14.33% (204/1424) of participants engaged with the program and contributed to 89.13% (2100/2356) of the total replies in TEXTMEDS. Most replies during TEXTMEDS were received during the middle of the program, with the least number shouldering this period in response to motivational and instructional message intents (Figure 1). In contrast, most replies during SupportMe/ITM were received at the beginning of the program (first 50 days) and in response to instructional and informative message intents (data not shown).

### ML Models to Classify Outgoing Message Intent and Reply Type

**Figure 2:**
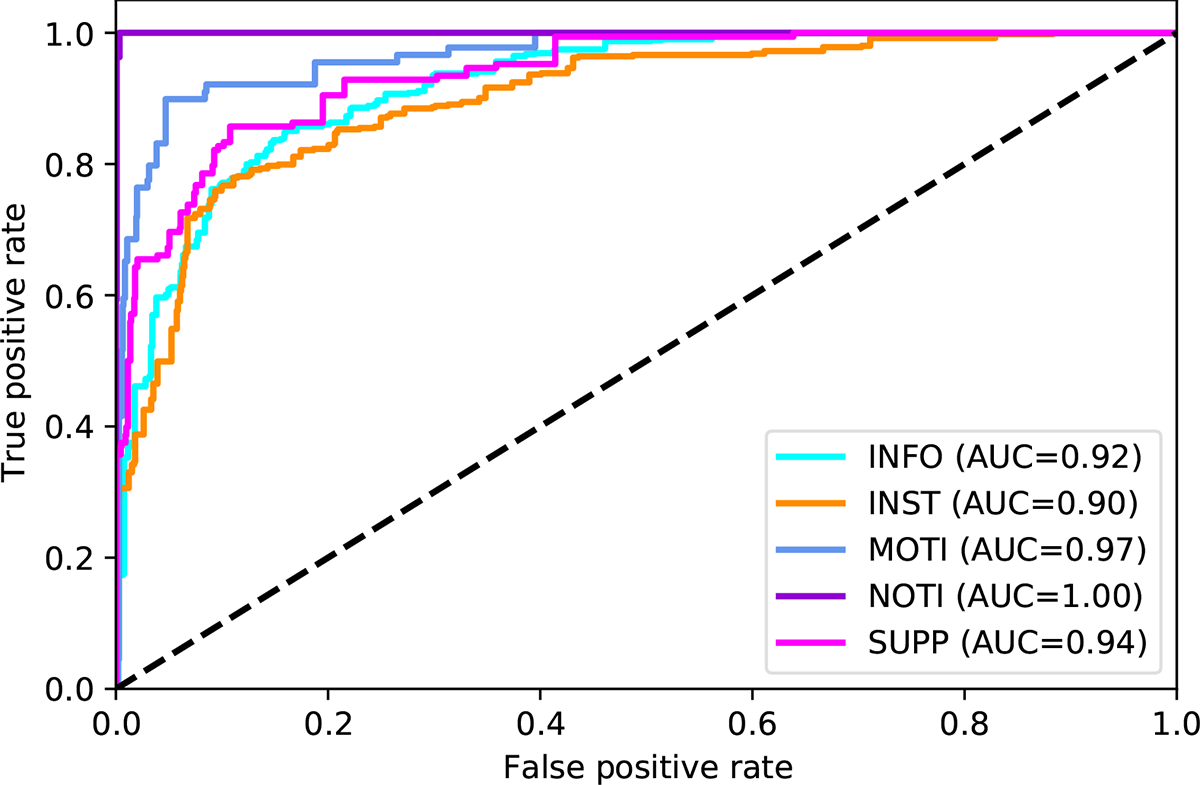
Receiver operating characteristic curves for predicting program message intent. Generated under one-vs-rest assumption (ie, each curve is generated assuming a binary scenario with the selected class against all other classes). AUC: area under the curve; INFO: Informative; INST: Instructional; MOTI: Motivational; NOTI: Notification; SUPP: Supportive.

Table 2 shows the classification report for the program messages according to message intent. Altogether, the ML model correctly classified the intent of program messages as 76.6% (95% CI 63.5%-89.8%; balanced accuracy) of the time (Table 2). Average specificity was 93.2%, positive predictive value 76.3%, and negative predictive value 93.4%. The average area under the curve from the ROC curves was 0.95 (Figure 2). Sensitivity was lowest for the *supportive* message intent (69.7%), and specificity was lowest for the *informative* message intent (86.8%).

**Table 2:** Machine learning performance for program message intent.

Message intent	Sensitivity (SD)	Specificity (SD)	PPV^a^ (SD)	NPV^b^ (SD)	FPR^c^ (SD)	FNR^d^ (SD)	F1-score (SD)
INFO^e^	0.797 (0.144)	0.868 (0.072)	0.850 (0.070)	0.840 (0.099)	0.132 (0.072)	0.203 (0.144)	0.815 (0.089)
INST^f^	0.761 (0.169)	0.885 (0.093)	0.795 (0.124)	0.887 (0.064)	0.115 (0.093)	0.239 (0.169)	0.759 (0.118)
MOTI^g^	0.778 (0.242)	0.968 (0.033)	0.671 (0.248)	0.986 (0.016)	0.032 (0.033)	0.222 (0.242)	0.702 (0.221)
NOTI^h^	0.800 (0.400)	0.999 (0.002)	0.900 (0.300)	0.994 (0.015)	0.001 (0.002)	0.200 (0.400)	1.000 (0.000)
SUPP^i^	0.697 (0.296)	0.940 (0.046)	0.635 (0.251)	0.962 (0.036)	0.060 (0.046)	0.303 (0.296)	0.741 (0.138)
Average^j^	0.766 (0.175)	0.932 (0.027)	0.763 (0.148)	0.934 (0.027)	0.068 (0.027)	0.234 (0.175)	0.782 (0.100)

^a^PPV: positive predictive value.

^b^NPV: negative predictive value.

^c^FPR: false positive rate.

^d^FNR: false negative rate.

^e^INFO: Informative.

^f^INST: Instructional.

^g^MOTI: Motivational.

^h^NOTI: Notification.

^i^SUPP: Supportive.

^j^Macroaveraged.

**Figure 3:**
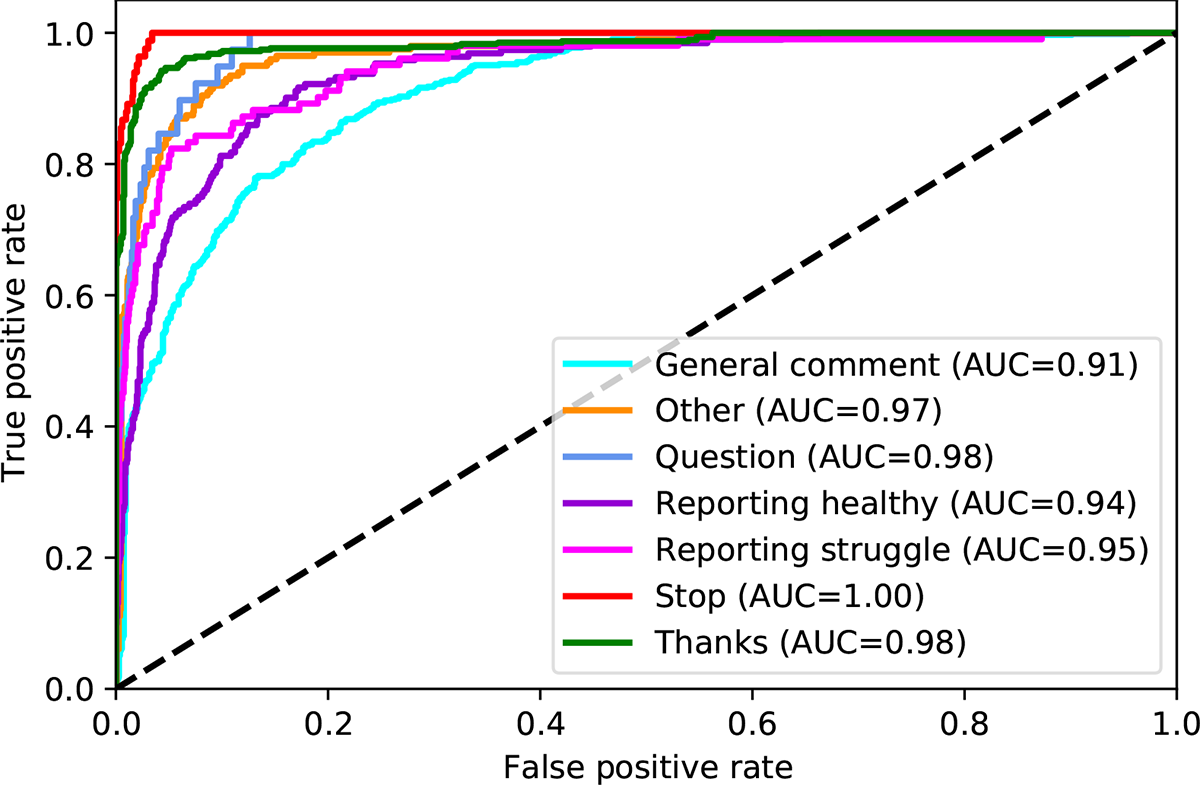
Receiver operating characteristic curves for predicting participant reply type. Generated under one-vs-rest assumption (ie, each curve is generated assuming a binary scenario with the selected category against all other categories). AUC: area under the curve.

Table 3 shows the classification report for the participant replies. Altogether, the ML model correctly categorized the replies with 77.8% (95% CI 74.1%-81.4%) balanced accuracy (Table 3). Average specificity was 95.7%, positive predictive value 72.6%, and negative predictive value 95.5%. Sensitivity was lowest with *reporting struggle* category (64.9%), and specificity was lowest with the *general comment* category (89.3%). The average area under the curve from the ROC curves was 0.96 (Figure 3).

**Table 3:** Machine learning performance for participant reply categories.

Participant replies	Sensitivity (SD)	Specificity (SD)	PPV^a^ (SD)	NPV^b^ (SD)	FPR^c^ (SD)	FNR^d^ (SD)	F1-score (SD)
General comment	0.684 (0.121)	0.893 (0.055)	0.817 (0.074)	0.815 (0.073)	0.107 (0.055)	0.316 (0.121)	0.737 (0.079)
Thanks	0.911 (0.050)	0.959 (0.026)	0.863 (0.090)	0.972 (0.027)	0.041 (0.026)	0.089 (0.050)	0.771 (0.099)
Question	0.815 (0.213)	0.976 (0.014)	0.474 (0.157)	0.995 (0.007)	0.024 (0.014)	0.185 (0.213)	0.592 (0.174)
Reporting healthy	0.707 (0.097)	0.940 (0.037)	0.601 (0.198)	0.960 (0.040)	0.060 (0.037)	0.293 (0.097)	0.623 (0.111)
Reporting struggle	0.649 (0.167)	0.979 (0.012)	0.696 (0.136)	0.976 (0.013)	0.021 (0.012)	0.351 (0.167)	0.658 (0.106)
Stop	0.860 (0.147)	0.993 (0.008)	0.888 (0.131)	0.992 (0.009)	0.007 (0.008)	0.140 (0.147)	0.866 (0.116)
Other	0.818 (0.082)	0.956 (0.029)	0.740 (0.132)	0.972 (0.016)	0.044 (0.029)	0.182 (0.082)	0.885 (0.065)
Average^e^	0.778 (0.048)	0.957 (0.012)	0.726 (0.071)	0.955 (0.013)	0.043 (0.012)	0.222 (0.048)	0.733 (0.054)

^a^PPV: positive predictive value.

^b^NPV: negative predictive value.

^c^FPR: false positive rate.

^d^FNR: false negative rate.

^e^Macroaveraged.

### Associations of Message Characteristics to Premature Program Stopping and Engagement

Overall, *supportive* message intent was associated with a reduced chance of premature program stopping (odds ratio [OR] 0.53, 95% CI 0.35-0.81; *P*=.003). For SupportMe/ITM, participants were less likely to reply *stop* following *informative* messages (OR 0.35, 95% CI 0.20-0.60; *P*<.001) and more likely to reply *stop* following *notification* messages (OR 5.76, 95% CI 3.66-9.06; *P*<.001). This differed from TEXTMEDS, where there was no significant association with *informative* (OR 1.25, 95% CI 0.78-2.02; *P*=.35) and *notification* messages (OR 1.89, 95% CI 0.95-3.74; *P*=.07; Table 4).

**Table 4:** Univariate logistic regression with program message intent and outcome variables (premature program stopping and engagement).

Message intent, outcome variables		Total				SupportMe/ITM				TEXTMEDS^a^				*P* value for interaction^b^
		OR^c^ (95% CI)	β coefficient	*P* value	OR (95% CI)		β coefficient	*P* value	OR (96% CI)		β coefficient	*P* value		
**Premature program stopping (ie, reply type “stop”)**														
	INFO^d^	0.69 (0.49-0.98)	–0.37	.04	0.35 (0.20-0.60)		–1.05	<.001	1.25 (0.78-2.02)		0.23	.35	<.001	
	INST^e^	0.98 (0.69-1.40)	–0.02	.93	0.86 (0.54-1.38)		–0.15	.54	1.03 (0.60-1.76)		0.03	.92	.63	
	SUPP^f^	0.53 (0.35-0.81)	–0.64	.003	0.60 (0.29-1.26)		–0.51	.18	0.58 (0.34-0.99)		–0.54	.05	.94	
	MOTI^g^	1.00 (0.52-1.91)	–0.00	.99	1.08 (0.43-2.73)		0.08	.87	0.98 (0.39-2.47)		–0.02	.97	.89	
	NOTI^h^	4.01 (2.80-5.75)	1.39	<.001	5.76 (3.66-9.06)		1.75	<.001	1.89 (0.95-3.74)		0.64	.07	.01	
**Engagement (ie, 3 replies and did not prematurely stop the program)**														
	INFO	1.76 (1.46-2.12)	0.56	<.001	2.16 (1.67-2.78)		0.77	<.001	1.62 (1.21-2.16)		0.48	<.001	.14	
	INST	1.47 (1.21-1.80)	0.39	<.001	1.68 (1.29-2.18)		0.52	<.001	1.51 (1.10-2.08)		0.42	.01	.63	
	SUPP	1.22 (1.01-1.49)	0.20	.04	1.77 (1.21-2.58)		0.57	.003	0.77 (0.60-0.98)		–0.27	.04	<.001	
	MOTI	1.18 (0.82-1.70)	0.17	.37	0.82 (0.50-1.34)		–0.20	.43	1.64 (0.92-2.93)		0.50	.10	.07	
	NOTI	0.14 (0.11-0.17)	–1.99	<.001	0.07 (0.05-0.10)		–2.61	<.001	0.28 (0.20-0.39)		–1.28	<.001	<.001	

^a^TEXTMEDS: Text Messages to Improve Medication Adherence and Secondary Prevention.

^b^*P* interaction refers to the comparison of the associations between each message intent with program type (SupportMe/ITM vs TEXTMEDS) and adjusted for message intent.

^c^OR: odds ratio.

^d^INFO: Informative.

^e^INST: Instructional.

^f^SUPP: Supportive.

^g^MOTI: Motivational.

^h^NOTI: Notification.

Overall, *informative* (OR 1.76, 95% CI 1.46-2.12; *P*<.001) and *instructional* (OR 1.47, 95% CI 1.21-1.80; *P*<.001) message intents were associated with increased engagement but not *motivational* (OR 1.18, 95% CI 0.82-1.70; *P*=.37) message intent (Table 4). *Supportive* message intent was associated with increased engagement in SupportMe/ITM (OR 1.77, 95% CI 1.21-2.58; *P*=.003) but reduced engagement in TEXTMEDS (OR 0.77, 95% CI 0.60-0.98; *P*=.04; for interaction, *P*<.001). *Notification* messages were associated with reduced engagement in both SupportMe/ITM (OR 0.07, 95% CI 0.05-0.10; *P*<.001) and TEXTMEDS (OR 0.28, 95% CI 0.20-0.39; *P*<.001); however, the strength of the association was greater for SupportMe/ITM (for interaction, *P*<.001).

## Discussion

### Principal Findings

In this study, ML models were created to categorize program message intent and participant replies from SMS text message–based programs with good accuracy. Thus, they can enable the monitoring and detailed characterization of program messages and participant replies, which can be used to further customize SMS text message–based programs. Furthermore, this study found that program message type can influence premature program discontinuation and encourage participant engagement. However, some of these associations varied or were attenuated by program type. This suggests that participant engagement may be maximized by adjusting program message characteristics, that program type and patient population type are important to consider, and that larger studies to examine these interactions will enable further program refinement.

We have previously assessed the accuracy of ML to *triage* participant text replies in an attempt to identify those requiring health professional review with only 1.4% false negatives and categorized replies according to *themes* with modest accuracy (0.723 weighted average accuracy) [20]. However, unlike this study, this was done without knowledge of the message sent that the participant is replying to or its intent. This limits the accuracy and contextualization of the reply modeling, and thus no conclusion regarding the characteristics of texts that elicit replies can be drawn. In this study, by contextualizing the participant replies to the program messages, we allowed a detailed assessment of which program message characteristics are likely to elicit different types of replies. Furthermore, the use of a *reporting struggle* category in this study, in addition to a *reporting healthy* category, which has been contextualized with the program message intent, allows the automated identification of participants who may need more or less tailored support, respectively.

A recent review of systematic reviews identified 3 reviews and 10 studies (clinical trials and feasibility studies) that measured engagement [25]. Of the included studies, only 1 quantified engagement with an SMS text message–based intervention, and the measure of engagement was the frequency of replies to reminders for blood glucose measurement [25]. There are no SMS text message–based studies reporting engagement in a population with CVD. Smartphone- and internet-based programs were the most common mHealth types in the studies assessing engagement, and although there was variability in the measure of engagement used, most measures focused on the way users interact with the program (eg, number of visits to a web-based program or frequency of responses). In this study, participants were more likely to engage with *informative* and *instructional* program message intents. These message intents use the behavior change techniques of goal setting and self-monitoring, both of which have been associated with higher engagement with digital health interventions (DHIs) [12,26]. Importantly, the pattern of participant interaction throughout the course of a DHI is dynamic and involves different levels of use over time [27]. Highlighting this in our study, the type of replies and message intent prompting replies changed throughout the duration of the TEXTMEDS program (Figure 1). In contrast to our study, a mixed methods study assessed the utility of either email or text prompts in encouraging engagement (as measured by the number of log-ins) in a web-based intervention for diabetes and found that email prompts increased engagement but not text prompts, and this was affected by email content [28]. However, over a 15-month period, only 7 text prompts were sent compared with 49 email prompts, which may explain the lack of significance.

Features enabling remote contact with a health care professional can positively influence engagement with DHIs [12,29,30], as can interactivity [31]. In this study, the associations between some message intents and premature program stopping or engagement differed by program type. For instance, with TEXTMEDS, *notification* messages were not associated with premature program termination, unlike SupportMe/ITM. In addition, the odds of *notification* messages decreasing engagement, although being elevated in both programs, were significantly higher in SupportMe/ITM than in TEXTMEDS (Table 4). These differences may be related to the encouraged flow of information—2-way communication was encouraged with TEXTMEDS (and with health counselor support) and not encouraged with SupportMe/ITM. Supporting this, Redfern et al [32] demonstrated, using qualitative methods, including reviewing user surveys and focus groups, that support felt from program participation and from health staff are important factors that influence engagement. Differences in patient populations may also explain the interaction effects with program type. Participants in SupportMe/ITM had stable chronic diseases compared with TEXTMEDS, where participants were recruited following an acute coronary syndrome, and it is reasonable to suspect that participants who were recruited in the context of symptoms may engage differently compared with participants without symptoms. This is consistent with Redfern et al [32], who also determined that initiating the mHealth program close to the time of a cardiovascular event was associated with increased engagement.

Unexpectedly, *supportive* messages were associated with decreased engagement in TEXTMEDS but increased engagement in SupportMe/ITM. However, the highest proportion of responses overall were elicited following *supportive* messages in TEXTMEDS and *informative* messages in SupportMe/ITM (Multimedia Appendix 1). Furthermore, TEXTMEDS was twice as long as SupportMe/ITM, and most of the responses received were in the first half of the program (Figure 1). Thus, it is possible that engagement decreased in the second half of the program and differentially affected supportive message intent compared with the others, as a larger number of similar messages may have been sent to participants and considered repetitive over the longer program duration.

### Clinical Implications

The results of this study contribute to the field of SMS text message–based interventions by (1) demonstrating that using ML can automatically and accurately categorize SMS text messages sent to and from participants in an SMS text message–based program to support their health, and (2) providing new knowledge on how participants engage with SMS text messages and factors associated with engagement and premature program termination. Overall, our ML models for characterizing program message intent and user replies enable the ability to monitor and describe the way participants interact with different SMS text message–based prevention programs. This has implications for the optimized development of future SMS text message–based programs, as the results suggest that participant engagement may be maximized (and premature program termination avoided) by adjusting message characteristics, that is, the clinical implications of message content affecting participant withdrawal and engagement are the potential of using this knowledge to alter future messages automatically in real time to sustain engagement throughout the intervention duration. This could minimize participant withdrawal and maximize the likelihood of behavior change. This has not been assessed in previous studies, and a lack of knowledge of participant-program interactions has limited the utility of existing SMS text message–based programs.

As there may be differences in the degree of interactivity encouraged (ie, 1-way vs 2-way communication), when assessing engagement and premature program stopping, we performed an interaction analysis (in addition to analyzing the programs separately and combined) to assess if the associations were affected by program type (Table 4). From these results, it is clear that program type can affect engagement. This is an important finding as there is large heterogeneity in existing SMS text message–based programs with respect to the degree of interactivity allowed, and recognizing that participants may interact differently with 1-way versus 2-way programs also potentially informs different approaches to optimizing engagement, depending on the program type.

Validation of these models with different SMS text message–based programs delivered to different population groups (different clinical and geographical settings across high-, middle-, and low-income countries) would assist in increasing generalizability and utility. Future research should explore the association between program engagement and intervention success or behavioral change. In addition, there is a need to determine whether modifying message characteristics can maximize participant engagement and whether this can lead to sustained behavior change.

### Limitations

Although manual categorization was done by health professionals, it is entirely possible that manual categorization may have differed if performed by a different group and, thus, affected the final ML models. In addition, some of the SMS text messages could be categorized into more than one category, which can also affect the final model; however, to minimize bias, we selected the majority category within each group of similar messages. Although there were differences in the populations between studies (Table 1), message content was similar between programs, allowing the clustering of messages into similar categories as described in the methods. Furthermore, although there was good overall accuracy with the ML models, the number of program messages and participant replies was relatively small for ML modeling, and the accuracy could be further improved by using a larger sample. This emphasizes the importance of validating these models against larger data sets.

As discussed in the *Methods* section, it was necessary to combine all studies to develop ML models to increase the accuracy of the ML models. Although there were differences in the clinical setting that the patients across the studies were recruited in (TEXTMEDS from hospital, ITM from community, and SupportMe from community and hospital), all patients had chronic diseases and were managed in the chronic phase of their illness (if recruited from the hospital, the messages were not initiated until the patient was discharged into the community). Thus, we do not believe that this would be a significant confounding variable. However, as discussed above, patients in TEXTMEDS were recruited following a symptomatic episode, which can affect engagement [32]. In addition, although all studies recruited patients with CVD, 2 of them (SupportMe and ITM) recruited patients with diabetes and chronic respiratory disease, and it is possible that the type of chronic disease influences engagement. Although all patients were recruited in the chronic phase of their illness, illness severity was not assessed. Although not previously assessed in the context of mHealth, there is survey evidence that severity of chronic diseases can affect perceived health attitudes [33]; thus, if applied to mHealth, it is possible that illness severity can affect engagement. Thus, the validation of these models against different types of chronic illnesses in future research is prudent.

This study compared a measure of engagement between 2 SMS text message–based programs that differ in the encouraged level of interaction (ie, 2-way vs 1-way communication). Thus, it is possible that participants replied depending on whether they were encouraged or not, and it is also possible that participants who did not engage with messages using our definition engaged with behavior change. However, almost one-third of the participants in SupportMe/ITM replied compared with one-fifth in TEXTMEDS, and, thus, using the frequency of replies (and excluding withdrawals) as a surrogate marker of engagement is a reasonable method of quantifying engagement, which is consistent with previous studies [25]. It is interesting that the proportion of participants who replied in SupportMe/ITM (1-way) is comparable with TEXTMEDS (2-way). In both types of programs, all messages were monitored by a health professional, and the patients were informed of this. Thus, although patients were not encouraged to reply by recruitment staff, we believe they did as they felt their replies would still be seen (even if they were not answered). Many of the texts (as seen in Textbox 1) would ask questions regardless of whether the program was 1-way or 2-way, and although these were meant to be rhetorical, participants replied. The major benefit of our definition of engagement is that we provide a simple quantifiable marker of engagement that can potentially be used across different SMS text message–based programs, allowing comparisons between studies. As TEXTMEDS was twice as long as SupportMe/ITM, participants had more opportunities to become engaged according to our definition, and this may explain the numerically higher proportion of participants who were engaged in TEXTMEDS compared with SupportMe/ITM (204/1424, 14.33% vs 98/1218, 8.05%).

The association between participant sociodemographics and engagement with DHIs and behavior change was outside the scope of this study and should be explored in future research. The timing of messages sent and message length may affect engagement but was outside the scope of this study and will be assessed in future research.

### Conclusions

In our study, using ML, we categorized outgoing and incoming messages from different SMS text message–based programs to support people with chronic diseases with good accuracy, enabling monitoring and detailed characterization of program messages and participant replies. Message intent can influence adherence (ie, not stopping the intervention) and participant engagement, although we suspect this association is affected by the type of interaction encouraged (ie, 1-way vs 2-way communication) and possibly the setting in which participants are recruited. The clinical implications include optimization of future SMS text message–based programs by using program message characteristics that maximize participant engagement and potentially behavior change.

## Appendix

Multimedia Appendix 1 Distribution of participant replies for each program message intent for TEXTMEDS (Text Messages to Improve Medication Adherence and Secondary Prevention) and SupportMe/ITM.

Multimedia Appendix 2 Distribution of response categories for TEXTMEDS (Text Messages to Improve Medication Adherence and Secondary Prevention) and SupportMe/ITM.

Multimedia Appendix 3 Participant reply category by program message intent for SupportMe/ITM.

Multimedia Appendix 4 Participant reply category by program message intent for TEXTMEDS (Text Messages to Improve Medication Adherence and Secondary Prevention).

## Abbreviations

CVD: cardiovascular disease
DHI: digital health intervention
mHealth: mobile health
ML: machine learning
NHMRC: National Health and Medical Research Council
OR: odds ratio
RCT: randomized controlled trial
ROC: receiver operating characteristic
TEXTMEDS: Text Messages to Improve Medication Adherence in Secondary Prevention
