# Supplementary figures and images for: Text Message Analysis Using Machine Learning to Assess Predictors of Engagement With Mobile Health Chronic Disease Prevention Programs: Content Analysis
Source: JMIR Mhealth Uhealth. 2021 Nov 10;9(11):e27779. doi: 10.2196/27779 (PMC8663456; doi:10.2196/27779)

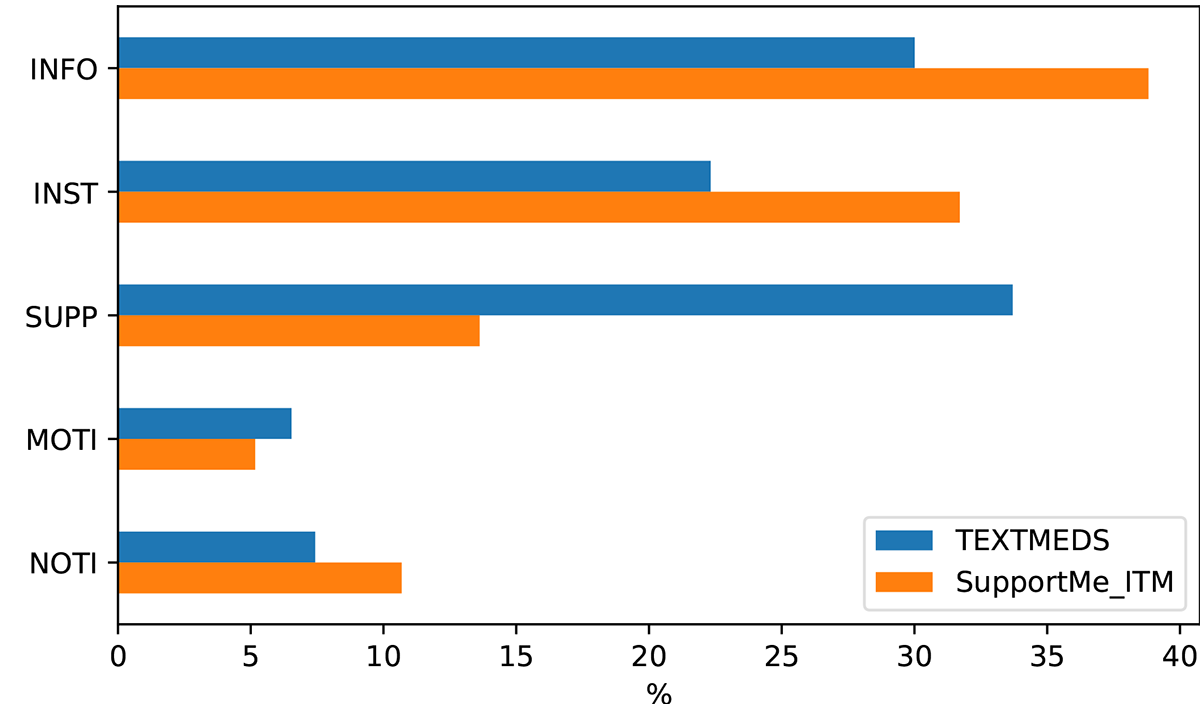

Supplement: Multimedia Appendix 1 [file mhealth_v9i11e27779_app1.png]

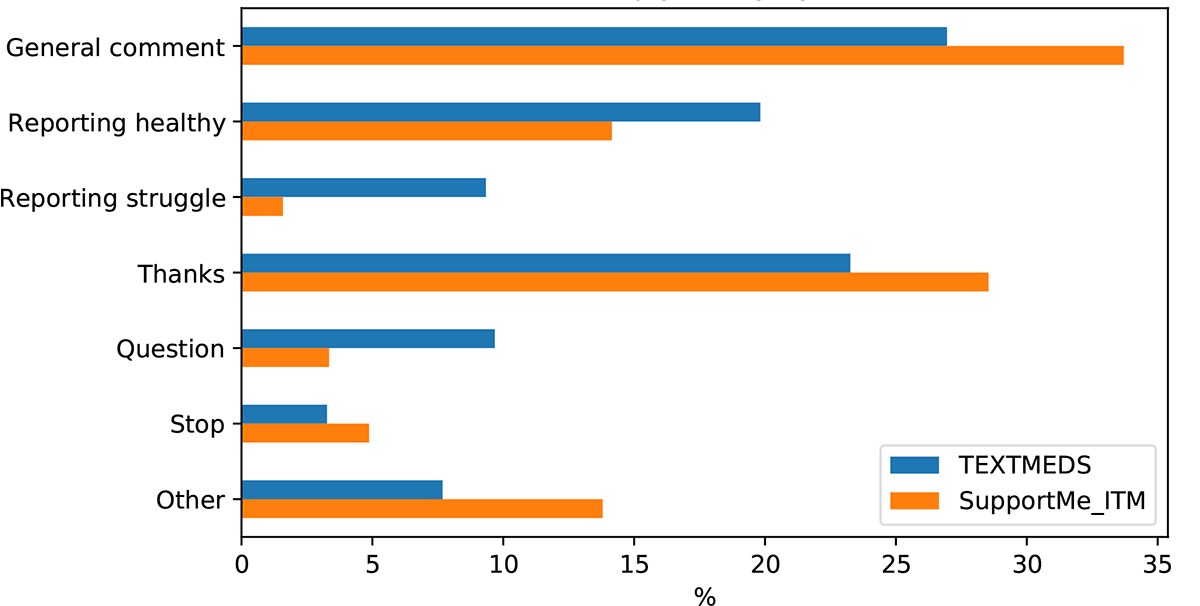

Supplement: Multimedia Appendix 2 [file mhealth_v9i11e27779_app2.png]
