# Supplementary material for: Text Message Analysis Using Machine Learning to Assess Predictors of Engagement With Mobile Health Chronic Disease Prevention Programs: Content Analysis
Source: JMIR Mhealth Uhealth. 2021 Nov 10;9(11):e27779. doi: 10.2196/27779 (PMC8663456; doi:10.2196/27779)
Supplement: Multimedia Appendix 3 [file mhealth_v9i11e27779_app3.docx]

## Multimedia Appendix 3

**Table S1: Participant reply category by program message intent for SupportMe/ITM**

| **Message Intent** | **General**  **comment** | **Question** | **Reporting healthy** | **Reporting struggle** | **Stop** | **Thanks** | **Other** | **TOTAL** |
| --- | --- | --- | --- | --- | --- | --- | --- | --- |
| **INFO** | 226 | 18 | 102 | 11 | 17 | 197 | 90 | 661 |
| **INST** | 176 | 19 | 88 | 7 | 26 | 140 | 84 | 540 |
| **SUPP** | 66 | 7 | 32 | 8 | 8 | 79 | 32 | 232 |
| **MOTI** | 40 | 2 | 17 | 0 | 5 | 18 | 6 | 88 |
| **NOTI** | 68 | 11 | 2 | 1 | 35 | 53 | 24 | 194 |
| **TOTAL** | 574 | 57 | 241 | 27 | 83 | 486 | 235 | 1715 |

INFO, Informative; INST, Instructional; MOTI, Motivational; NOTI, Notification; SUPP, Supportive
