# Supplementary material for: Text Message Analysis Using Machine Learning to Assess Predictors of Engagement With Mobile Health Chronic Disease Prevention Programs: Content Analysis
Source: JMIR Mhealth Uhealth. 2021 Nov 10;9(11):e27779. doi: 10.2196/27779 (PMC8663456; doi:10.2196/27779)
Supplement: Multimedia Appendix 4 [file mhealth_v9i11e27779_app4.docx]

## Multimedia Appendix 4

**Table S2: Participant reply category by program message intent for TEXTMEDS**

| **Message Intent** | **General**  **comment** | **Question** | **Reporting**  **healthy** | **Reporting**  **struggle** | **Stop** | **Thanks** | **Other** | **TOTAL** |
| --- | --- | --- | --- | --- | --- | --- | --- | --- |
| **INFO** | 198 | 73 | 118 | 56 | 27 | 163 | 72 | 707 |
| **INST** | 192 | 48 | 108 | 31 | 18 | 83 | 46 | 526 |
| **SUPP** | 162 | 101 | 211 | 118 | 18 | 143 | 41 | 794 |
| **MOTI** | 70 | 4 | 27 | 9 | 5 | 28 | 11 | 154 |
| **NOTI** | 13 | 2 | 3 | 6 | 9 | 131 | 11 | 175 |
| **TOTAL** | 635 | 228 | 467 | 220 | 77 | 548 | 181 | 2356 |

INFO, Informative; INST, Instructional; MOTI, Motivational; NOTI, Notification; SUPP, Supportive
